# Tools for Assessing Translation in *Cryptococcus neoformans*

**DOI:** 10.3390/jof7030159

**Published:** 2021-02-24

**Authors:** Corey M. Knowles, Kelcy M. McIntyre, John C. Panepinto

**Affiliations:** Department of Microbiology and Immunology, Jacobs School of Medicine and Biomedical Sciences, University at Buffalo, Buffalo, NY 14201, USA; coreykno@buffalo.edu (C.M.K.); kelcymci@buffalo.edu (K.M.M.)

**Keywords:** translation, initiation, elongation, repression, translation regulation, host adaptation

## Abstract

*Cryptococcus neoformans* is a ubiquitous environmental fungus capable of establishing an infection in a human host. Rapid changes in environments and exposure to the host immune system results in a significant amount of cellular stress, which is effectively combated at the level of translatome reprogramming. Repression of translation following stress allows for the specific reallocation of limited resources. Understanding the mechanisms involved in regulating translation in *C. neoformans* during host infection is critical in the development of new antifungal drugs. In this review, we discuss the main tools available for assessing changes in translation state and translational output during cellular stress.

## 1. Introduction

*Cryptococcus neoformans* is a human pathogen and a ubiquitous environmental fungus that must quickly adapt from living in its environmental niche to surviving inside of the human lung. Upon inhalation by the human host, *C. neoformans* encounters cellular perturbations in the form of mammalian core body temperature, oxidative stress from oxidative bursts generated by resident lung macrophages, and nutrient deprivation due to tissue sequestration of usable carbon sources and trace minerals [1]

In order to adapt to the environment inside of the human host, one of the tools *C. neoformans* employs is rapid translational reprogramming, which allows for the reallocation of valuable energetic resources to counter this suite of stressors [2,3]. Reprogramming is characterized by a repression of protein synthesis, which is often proportional to the degree of the stress. Understanding how *C. neoformans* is able to use translational regulation to rapidly adapt to such stress gives us valuable insight into how it is able to be a successful human pathogen. In this review, we will provide an overview of the processes governing translation in eukaryotes, how these processes are regulated in response to stress, and the methods that we can employ in the *Cryptococcus* system to investigate translational regulation in this important pathogen.

### 1.1. Translation Initiation

**Figure 1:**
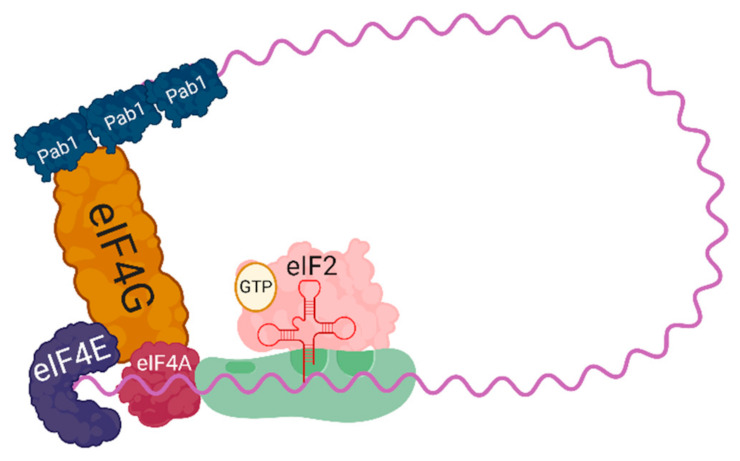
A schematic of a 43S preinitiation complex (the 40S small ribosomal subunit with eIF2 bound to GTP, and Met-tRNA^Met^) loaded onto an mRNA, bound by eIF4F (composed of the cap binding protein eIF4E, the helicase eIF4A, and the scaffolding protein eIF4G). The scaffolding protein eIF4G spans to bind the poly-A binding protein, Pab1, resulting in circularization of the translational complex.

Translation can be divided into three stages; (1) initiation (2) elongation (3) termination. Initiation has long been held as the rate-limiting step of protein translation and is heavily influenced by factors that perturb cellular homeostasis. A complete translating ribosome is comprised of one large ribosomal subunit and one small ribosomal subunit, 60S and 40S, respectively [4,5]. Before the 60S subunit can join the 40S subunit and begin translation elongation, the 40S subunit must join with the necessary initiation factors at the 5′ cap of a fully mature messenger RNA forming the 43S preinitiation complex (PIC) (Figure 1). Assembling the 43S PIC begins with the joining of the ternary complex, consisting of eIF2-GTP and a Met-tRNA^Met^, with a 40S subunit. The 43S PIC is then is able to bind to the 5′ cap through interactions with eIF4F, which is comprised of the helicase eIF4A, the scaffolding protein eIF4G, and the cap binding protein eIF4E. Once bound to the cap, the 43S PIC is able to begin scanning the mRNA for a start codon to pair with the tRNA^Met^. After a start codon with sufficient Kozak context is recognized, the GTP bound to eIF2 is hydrolyzed and the 60S subunit is able to join, completing a translationally competent 80S ribosome. This process of start codon identification and subunit joining comprises the initiation step of translation.

### 1.2. Translation Elongation

The result of initiation is an active ribosome with compartmentalized functions that is able to decode the mRNA three nucleotides at a time. The compartments are defined by the 40S subunit and are named after their particular role. They are the acceptor (A), peptidyl (P), and exit (E) sites. The acceptor (A) site is positioned over the 3′ most end of the ribosome and accepts the tRNA that is antisense to the underlying cognate codon in the mRNA. The eukaryotic elongation factor 1A (eEF1A) directs aminoacyl t-tRNAs into the A site in a GTP-dependent manner and upon pairing of the correct tRNA, eIF1A triggers the hydrolysis of GTP allowing the aminoacyl-tRNA to enter the empty A site (Reviewed in [4,5]). The true enzymatic function of the ribosome occurs at the P site, where a peptide bond is quickly formed between the amino acids occupying the A and P sites through the catalytic activity of the 28S rRNA. The position of a particular codon within the ribosome is changed through the action of eEF2, which hydrolyzes GTP to ratchet the mRNA through one codon at a time [6]. This translocation event places the now deacylated tRNA from the P site into the E site, while allowing the A site to be occupied by a new codon. Once a deacylated tRNA occupies the E site and a new aminoacylated tRNA is accepted at the A site, the deacylated tRNA in the E site is released [7]. Unlike mammalian systems, *C. neoformans* and other unicellular eukaryotes also require an additional elongation factor, the ATPase eEF3, which has been shown to span the ribosome from the A to E sites, and assist in tRNA release [8]. Elongation proceeds in such a fashion until a codon is reached where no corresponding tRNA exists. At this point, termination is initiated, and ribosome recycling begins.

### 1.3. Regulation of Initiation and Elongation

Programmed, systematic regulation of translation in response to stress can occur at both the levels of initiation and elongation, primarily by limiting the availability of necessary factors for each step to occur. At the level of initiation, this is seen as the phosphorylation of the α subunit of eIF2 resulting in reduced ternary complex formation, and subsequently, a loss of translation initiation [5,9]. In *C. neoformans*, the sole kinase responsible for phosphorylation of eIF2α is encoded by *GCN2*. Deletion of *GCN2* results in sensitivity to oxidative stress, and a failure to repress translation initiation [10]. Gcn2 has canonically been shown to be activated during amino acid starvation through the binding of uncharged tRNA, but more recent evidence shows that ribosome collision during stress can also result in Gcn2 activation in mammalian systems and *Saccharomyces cerevisiae* [11,12,13].

Translation can also be regulated at the level of elongation through phosphorylation of eEF2. The conserved mechanism of eEF2 phosphorylation results in an eEF2 which is allosterically unable to bind to the ribosome and facilitate ribosome translocation [14]. As a result of eEF2 phosphorylation, translation elongation is repressed globally. An eEF2 kinase has been identified in mammalian systems and other yeast models, but the *C. neoformans* eEF2 kinase is yet to be identified. Our laboratory is currently investigating the role of eEF2 in the stress adaptation response and seeking to identify the *C. neoformans* eEF2 kinase.

Previous work by our laboratory has shown that *C. neoformans* mutants that are unable to repress translation, both a *gcn2*Δ strain, and a strain lacking the major mRNA deadenylase Ccr4, are unable to respond and adapt to physiologically relevant stresses such as host temperature and oxidative stress [3,10]. In addition, a *ccr4*Δ mutant exhibits decreased virulence in a mouse model [15]. These data together inform us that the ability of *C. neoformans* to repress translation and undergo translational reprogramming is crucial for its ability to be a successful pathogen, and targeting translation machinery with novel small molecules is a viable route in developing new chemotherapeutic treatments against *C. neoformans.*

## 2. Methods Used in Investigating Translation

As methods to assess the transcriptome, such as RNA sequencing, have become less expensive, easier to perform, and with increased depth, we have gained access to an immense amount of data pertaining to mRNA abundance. While this data provides us with insight to how changes are being affected at the mRNA level, it is known that the transcriptome, or mRNA abundance, does not correlate with the proteome, suggesting that translational regulation greatly contributes to achieving a stress adaptive proteome [16]. The use of translational inhibitors, as well as tools to assess translational state and translational output, can illuminate the effect of gene mutation and experimental conditions on cellular translation in *C. neoformans*.

### 2.1. Translation Inhibitors

The ribosome is perhaps the best-exploited target of anti-infectives, as targeting protein synthesis is an effective way to halt microbial proliferation. The ribosome is the primary target of many classes of antibiotics, including the popular aminoglycosides, used to combat bacterial infections. Due to conservation amongst eukaryotic ribosomes, the current chemotherapeutic treatments that target the *C. neoformans* ribosome would result in toxicity to the human host. However, through the use of translation inhibiting antibiotics and small molecules in the laboratory, we are better able to characterize the *C. neoformans* translating ribosome and the ribosome associated factors necessary for translation. Translation inhibitors, which aid in these studies and are efficacious in *C. neoformans,* are the long used antibiotics cycloheximide and puromycin, as well as the more recently discovered class of small molecules, rocaglates, which have been shown to target fungal translation initiation [17].

#### 2.1.1. Cycloheximide

Cycloheximide is a translation inhibitor that functions at the level of elongation. It serves to block elongation by binding to the E site on 60S ribosomal subunits, subsequently inhibiting eEF2 mediated translocation [18,19]. While cycloheximide is an effective translation elongation inhibitor, after cycloheximide binds to the 60S subunit, the ribosome can still undergo one translocation event [4]. This characteristic of cycloheximide contributes to abnormalities seen when translational inhibitors are used during preparations of cells that are to be used in ribosome profiling experiments, and as such, should be avoided. Despite its ability to induce bias, cycloheximide is a powerful tool for studying translation when nucleotide resolution is not of concern. Experimentally, its primary function is to lock ribosomes into their place at the time of treatment, preventing ribosome run-off during sample preparation. Additionally, in *C. neoformans,* retention of ribosomes on mRNAs by cycloheximide protects mRNAs from degradation [10]. These combined attributes of cycloheximide contribute to the fidelity of polysome profiling. Cycloheximide can also be used as a negative control during translational output assays, such as the puromycin incorporation assay and fluorescent labeling through click chemistry, which are discussed below. At low concentrations, cycloheximide can be used to induce stalling of a small population of ribosomes, resulting in ribosome collisions, which can be visualized as disomes and trisomes by polysome profiling following RNaseI digestion [20].

#### 2.1.2. Puromycin

Another key translational inhibitor used in studying translation is puromycin, which is a naturally occurring antibiotic produced by *Streptomyces alboniger.* The structure of puromycin resembles an aminoacylated tRNA, allowing for its non-template-driven incorporation into the ribosome A site during translation [21]. Once puromycin enters the A site of the ribosome, a peptidyl-puromycin bond is formed, resulting in premature translation termination independent of translational termination machinery, release of the nascent puromycilated peptide, and splitting of the ribosome into its subunits [21,22,23]. These characteristics of puromycin’s action, both the release of a puromycilated nascent chain and the splitting and release of ribosomes, provide us with tools to assess *C. neoformans* translational activity, further explained below.

#### 2.1.3. Rocaglates

Rocaglates are molecules that have been shown to repress translation through inhibition of the eukaryotic initiation factor, eIF4A [24]. Rocaglates have been studied for their effects on cancer cells, and more recently have been shown to initiate cell death in *Candida auris* [17]. In this study, a library of rocaglate derivatives was screened to identify a compound with specificity toward *C. auris*, and the identified molecule exhibited limited activity toward *C. neoformans*. This work is fundamentally important, as it demonstrates that rocaglate drug development can be focused to target translation in fungi in a selective manner, and avoid host toxicity.

Two rocaglates, rocaglamide A (roc A) and silvestrol, have shown to repress translation through two slightly different mechanisms of action on eIF4A. Roc A causes eIF4A to clamp onto RNA purine-rich regions, stabilizing the eIF4A:RNA complex and blocking 43S PIC scanning [25]. The mechanism of action of Silvestrol is not related to purine-rich regions, but instead is correlated with the presence of G-quadruplexes [26]. Roc A and silvestrol have shown antiproliferative activity against many human cancer lines at nanomolar concentrations along with efficacy in in vivo experiments [27]. Rocaglates are promising molecules for repressing translation in *Cryptococcus neoformans* due to their repression of eIF4A. Minor decreases in total levels of eIF4A in yeast drastically decrease translation of all mRNAs [28], indicating that rocaglates targeting eIF4A in *Cryptococcus neoformans* may be effective in repressing translation.

The rocaglates rocaglamide A and silvestrol inhibit the growth of *C. neoformans* (McIntyre and Panepinto, unpublished data). Using a broth microdilution MIC analysis to assess growth inhibition, Roc A showed growth inhibition of *Cryptococcus neoformans* H99 at 25 and 50 μM, and growth inhibition of *Cryptococcus neoformans* JEC21 at 50 μM. Silvestrol showed growth inhibition of *Cryptococcus neoformans* H99 at 32 μM, and a dose-dependent growth inhibition of *Cryptococcus neoformans* JEC21 from 8 to 32 μM (data not shown). This class of small molecules will help further our understanding of the nuances of translation in *C. neoformans* and help identify new strain specific antifungal chemotherapeutics.

### 2.2. Polysome Profiling

**Figure 2:**
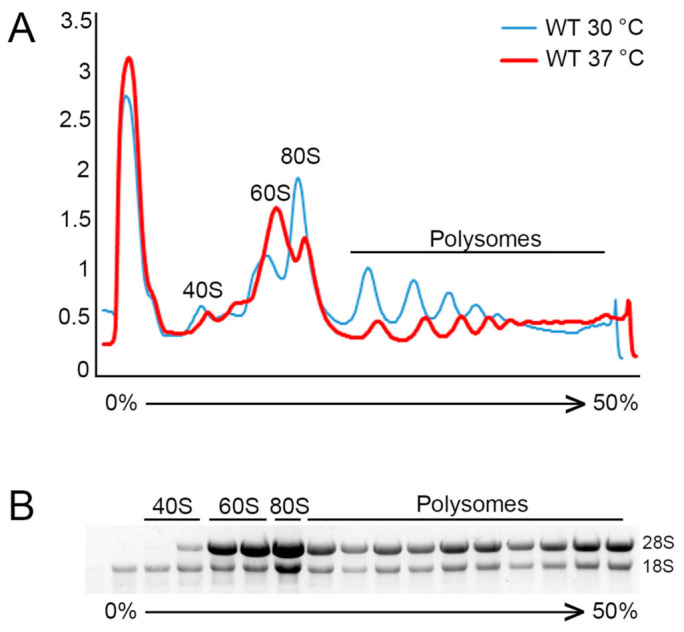
Polysome profiling and analysis. (**A**) Representative polysome traces for an unstressed *C. neoformans* culture (30° C) in blue, compared to a polysome profile subjected to temperature stress (37 °C) for 30 min. The decrease in area under both the polysome fraction and 80S monosome peak, along with an increase in the 60S subunit peak, is indicative of repressed translation, reduced initiation, and an increase in the free ribosome pool. (**B**) RNA following electrophoretic separation from isolated individual fractions from representative polysome profiles. Visible are fractions containing only 18S rRNA representing the small 40S ribosomal subunit, fractions with predominantly 28S rRNA representing the large 60S ribosomal subunits, a fraction containing a strong enrichment of 80S monosomes, and the polysome portion showing distribution of 80S ribosomes.

The molecular method of polysome profiling allows assessment of the translational state, visualizing the abundance of 40S and 60S ribosomal subunits, 80S monosomes, and polyribosomes, and their association with mRNAs. Polysome profiling relies on the difference in sedimentation coefficients of each of these translational components as they settle throughout a 10–50% sucrose gradient during ultracentrifugation. Once distributed throughout the gradient, the relative abundance of each of these populations is able to be determined based on the UV absorbance at 254 nm by the present RNA as the gradient is pumped out through a flow cell. The absorbance is then plotted for analysis as seen in Figure 2.

#### 2.2.1. Gradient Preparation

(1) Prepare 50 mL each of 10% and 50% sucrose solutions in the following buffer:
  Tris-HCl, pH 8: 20 mM
  KCl: 140 mM
  MgCl_2_: 5 mM
  DTT: 0.5 mM
  Cycloheximide: 0.1 mg/mL
  Heparin: 0.5 mg/mL
  Sucrose: 5 g and 25 g, respectively
(2) Use the Amersham gradient maker to make 10 mL gradients from 5 mL of 10% sucrose solution and 50% sucrose solution per manufacturer’s instructions. (Alternatively, in the absence of a gradient maker, layer 2 mL each of 10%, 20%, 30%, 40%, and 50% sucrose solutions and allow the gradient to equilibrate at 4°C overnight.)

Note: For comparable profiles, use gradients made on the same day from the same stock solutions.

#### 2.2.2. Culture Preparation

(1) Start cultures in 250 mL baffled flasks at OD_600_ = 0.15–0.20 in the desired media. A minimum volume of 50 mL is recommended. Incubate cultures, shaking, until midlogarithmic growth phase is reached, an OD_600_ = 0.55–0.70.
(2) If translation is to be assessed for response to stress or specific compounds, treat cultures appropriately ensuring a no stress/no drug control is also analyzed
(3) Pellet cells by centrifuging for 2 min at 4000 RPM and flash freeze cultures in liquid nitrogen to preserve ribosome position.

#### 2.2.3. Polysomes Extraction and Ultracentrifugation

(1) Prepare polysome lysis buffer and chill on ice:
  Tris-HCl, pH 8: 20 mM
  KCl: 140 mM
  MgCl_2_: 5 mM
  DTT: 0.5 mM
  Cycloheximide: 0.1 mg/mL
  Heparin: 0.5 mg/mL
(2) Thaw pellets on ice and resuspend in 5 mL lysis buffer. Transfer to a 14 mL snap-cap tube.
(3) Centrifuge at 4000 RPM for 5 min to pellet.
(4) Resuspend pellet in 1 mL lysis buffer and transfer to microfuge tube.
(5) Centrifuge at 4000 RPM for 5 min to pellet.
(6) Aspirate supernatant using a pipette.
(7) Resuspend pellet in 50 μL of lysis buffer.
(8) In an Eppendorf Safe-Lock tube, layer 0.5 mL of 0.5 mm glass disruption beads (RPI). Add resuspended pellet to the top of the beads, and layer with another 0.5 mL of beads.
(9) Lyse in Bullet Blender Tissue Homogenizer, chilled with dry ice, for 5 min on speed 12. (Alternatively, cells can be lysed by vortexing for 30 s, followed by 30 s of incubation on ice for a total of 5 times.)
(10) Add an additional 150 μL of lysis buffer to lysate and beads, and vortex to mix.
(11) Remove lysate from beads and transfer to a new microfuge tube, and centrifuge at 4 °C for 10 min at 15,000 RCF to clear lysate
(12) Transfer supernatant to new microfuge tube
(13) Quantify RNA using a Nanodrop spectrophotometer (or other suitable method).
(14) Layer an equivalent amount of lysate (based on RNA quantification) in equal volumes carefully on top of each gradient.
  - 100–250 μg if only a profile is needed.
  - Up to 350 μg if fractions will be collected for analysis of nucleic acids or protein.
(15) Centrifuge in an SW41 Rotor at 39,000 RPM for 2 h at 4 °C.

#### 2.2.4. Polysome Profile and Fraction Collection

(1) Prepare Isco UA-6 UV/VIS detector with 254 nm filter along with Isco Retriever 500 fraction collector (if fractions are to be collected) as per manufacturer’s instructions.
(2) Construct a Teledyne tube piercer with tubing connected to a peristaltic pump.
(3) Position the gradient in the tube piercer, pierce the tube, and pump the gradient (0.75 mL/min.) through the flow cell while reading absorbance at 254 nm.
(4) Simultaneously collect 500 μL fractions and denote the beginning and end of each fraction on the polysome profile trace.
  - If using the Isco UA-6 detector, the output can be digitally converted using a DATAQ DI-1110 data acquisition device and recorded using the WinDaq recording software.

#### 2.2.5. Protein and Nucleic Acid Extraction

(1) Precipitate RNA and protein complexes by adding three volumes of cold 95–100% ethanol to each fraction and precipitate at −80 °C overnight. (Alternatively, if only protein precipitation is required, 25% *w*/*v* TCA can be used in place of ethanol followed by three acetone washes.)
(2) Centrifuge the fractions at 15,000 RCF for 20 min at 4 °C
(3) Aspirate supernatant with a pipette.
(4) Quickly resuspend pellets in 250 μL RNase free water and immediately add 750 μL of TRIzol LS (ThermoFisher, New York, NY, USA). Proceed with manufacturer’s instructions to isolate RNA, protein, or both.

At the most basic level, analysis of polysome profiles can be performed by comparing profiles between two or more conditions, such as a wild type untreated versus a wild type treated sample, or a treated mutant sample versus its respective control. Overlaying the polysome profile traces will allow you to qualitatively visualize changes in subunit, monosome, or polysome peaks distribution (Figure 2A). Changes in the area under the curve of these peaks relative to another condition can provide insight into the translational state of the cells under a particular condition relative to each other. For example, a general reduction in the polysome portion of the plot combined with an increase in the subunit peaks is indicative of fewer ribosomes on mRNAs resulting in reduced translation. A redistribution pattern characterized by a decrease in polysomes with a corresponding increase in the 80S monosome peak likely indicates that translation is being repressed at the level of elongation, resulting in an increase in transcripts containing just a single ribosome. A polysome profile where there is an increase in the polysome portion tells us that there are in total more ribosomes on mRNA, potentially producing more overall protein product. These transcripts may contain more ribosomes because there is a higher translational demand. Alternatively, such a profile could also indicate a defect in ribosome release, causing a buildup of ribosomes on mRNAs.

Following trace acquisition, a researcher interested in the extent a particular RNA or protein is associated with translating ribosomes can collect samples fractionated by density for further processing. Following fractionation of the sucrose gradient, RNA and/or protein can be precipitated from each fraction, as outlined in the protocol above, and RNA electrophoresis, northern blotting, RT-qPCR, Western blotting, or even mass spectrometry can be performed. Electrophoretic separation of RNA isolated from individual fractions will allow you to see which fractions contain individual ribosomal subunits by the respective rRNA bands (Figure 2B). If you desire to see what portion of the polysome profile a particular mRNA is associating with, a northern blot can be performed for abundant transcripts, while RT-qPCR can be used for lowly expressed mRNAs. Our laboratory has previously performed RNA sequencing following polysome profile fractionation of cells stressed at 37 °C, allowing us to quantify the extensive mRNA decay dependent translatome reprogramming that occurs under this stress condition [29]. Likewise, protein can be isolated from each fraction and a western blot can be performed for a protein of interest to determine if it is differentially associated with the translational machinery during different treatments or conditions. On a large scale, mass spectrometry can be performed to assess all proteins associated with the translational machinery. This technique can be applied to identify novel proteins which may be associated with the ribosome to alter the translational state. Heat shock proteins, for example, have been shown to increase translation rates in yeast whereas components of cellular machinery involved in ribosome rescue, ribosome recycling, and mRNA decay, such as Hel2 and Cue2, repress translation [20,30,31,32,33,34].

Recently ribosome profiling, also referred to as ribosome sequencing, was performed in *C. neoformans* for the first time [35]. Ribosome profiling requires the digestion of all mRNA by RNaseI that is unprotected by either ribosomes or other RNA binding proteins, followed by size selection for isolation of ribosome protected fragments, and deep sequencing of these fragments [36]. This technique reveals the location of ribosomes across the transcriptome and allows for the measurement of genome wide translation and mRNA specific translational efficiency. With the inclusion of disome protected fragments, deep sequencing can also reveal the location of stalled translation and can identify specific mRNAs on which ribosomes are stalled [31,37,38]. Using disome analysis, our laboratory is currently working to determine the link between translation repression as seen by ribosome collision and translatome reprogramming.

When analyzing fractions collected from polysome profiles for either RNA or protein content, it is important to control for complexes which may not actually be ribosome associated, but rather co-sediment with ribosome complexes due to sedimentation coefficient similarities. One can control for this caveat by shifting the ribosome population to the less dense portion of the gradient to determine if the protein or RNA of interest tracks in a ribosome-dependent manner. This can be accomplished by running a parallel sample treated with EDTA, which chelates Mg^2+^ essential for 80S monosome maintenance [39,40].

Other considerations are to be made depending on the downstream application of the fractions after polysome profiling. In the instance of anything involving the use of reverse transcriptase, heparin should be avoided as it will inhibit the function of the enzyme [41,42]. The use of heparin during sample preparation is not necessary if proper RNA handling precautions are taken, but if the use of heparin is necessary, treatment of RNA samples with heparinase or precipitation of RNA with lithium chloride can be performed before the reverse transcription step. Additionally, if the samples are to be used for ribosome foot printing following RNaseI digestion, flash freezing should be used during sample collection instead of the addition of translation inhibitors, specifically cycloheximide, for preserving ribosome position on a transcript as cycloheximide has been shown to induce transcriptome wide bias in ribosome position [43,44,45,46].

### 2.3. Measuring Translational Output

Translational output is the bulk measure of protein that is being produced during a given time. This output is a function of ribosomes transiting mRNAs, and producing protein products. One of the largest caveats to assessing translation by polysome profiling is that while the technique allows us to generally and globally assess ribosomes present on mRNAs, it does not tell us if the ribosomes are actively translating. While a decrease in polysome peaks can definitively tell us that there are fewer ribosomes on mRNAs, and by consequence a decreased translational output, an increase or sustained polysome fraction does not necessarily correspond to increased translational output. This can be seen in cycloheximide-treated polysome profiles where polysomes are retained, but the ribosomes are locked in place by cycloheximide, producing no protein product. Treatment of *C. neoformans* with cycloheximide leads to a polysome profile indicative of robust translation, and stabilization of mRNAs associated with those ribosomes [10]. In the case of cycloheximide, ribosome translocation is inhibited. Intrinsic ribosome stalling can occur in response to physiologically relevant stressors such as heat shock and oxidative stress, and result in ribosome collision and activation of ribosome quality control [12,47,48,49,50]. An easy way to determine if there is major repression of translation is to measure bulk protein production.

Historically, measuring translational output is performed using ^35^S methionine incorporation and measurement by scintillation counting or gel exposure. Newer, non-radioactive methods allow easier, safer, and less expensive measurement of translational output, each with their own advantages and limitations. These include puromycin incorporation, which is an immunoblot based variation on the SUnSET assay, a method that relies on puromycin incorporation into nascent polypeptide chains [51,52], and homopropargylglycine (HPG) incorporation followed by CLICK chemistry fluorescent labeling and quantification of fluorescence [53,54].

#### 2.3.1. Puromycin Incorporation Assay

**Figure 3:**
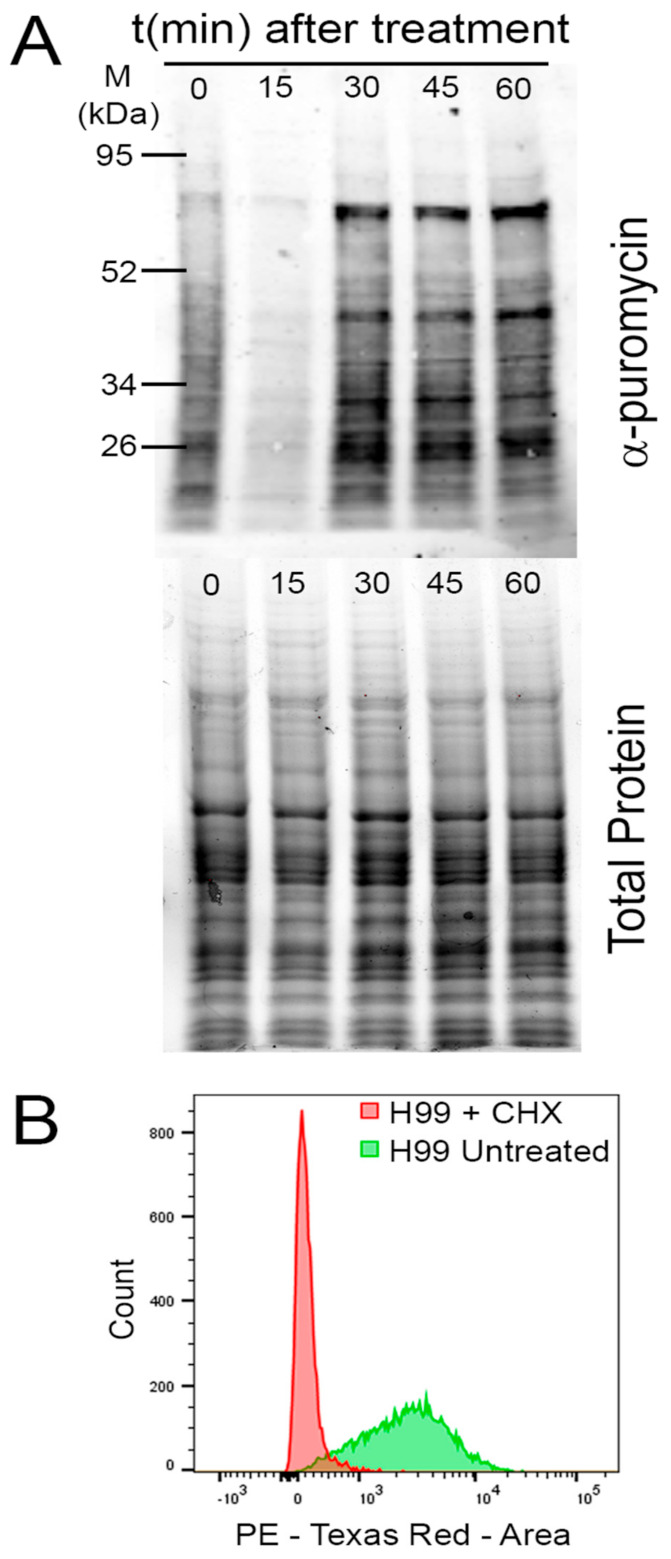
Measuring translational output. (**A**) Top panel: A western blot of whole lysates from representative samples treated with temperature stress for the indicated time, using an α-puromycin antibody to recognize puromycilated peptides, indicative of translational output. Bottom panel: Total protein for normalization of the α-puromycin signal. (**B**) A histogram of flow cytometry results following the click chemistry labeling reaction of incorporated HPG. The histogram in green shows basal levels of translational output while the histogram *n* red demonstrates inhibition of translational output by cycloheximide (CHX) treatment.

Treatment with sublethal concentrations of puromycin for a short time prior to harvesting of cells will result in a population of puromycilated polypeptides that are representative of the level of translational output during the given time [51]. A subsequent western blot using an anti-puromycin antibody was normalized to total loaded protein (Figure 3A).

Puromycin incorporation assay:

(1) Start 50 mL cultures in baffled flasks at OD_600_ = 0.15–0.20 in the desired media. Incubate cultures, shaking, until mid-logarithmic growth phase is reached, an OD_600_ = 0.55–0.70.
(2) If translation is to be assessed for response to stress or specific compounds, treat cultures appropriately ensuring a no-stress/no-drug control is also analyzed.
(3) 10 min prior to your desired time point, pellet cells for 2 min at 4000 RPM, resuspend in a volume of 5 mL of media (smaller volume used to limit the quantity of puromycin used) with a final concentration of 150 μg/mL.
(4) Allow puromycin to incorporate for 10 min.
(5) Pellet cells by centrifuging for 2 min at 4000 RPM and flash freeze cultures in liquid nitrogen.

Cell lysis:

(1) Thaw pellets on ice and resuspend pellet in 30 μL lysis buffer.
  HEPES pH 7.4: 15 mM
  KCl: 10 mM
  MgCl_2_: 5 mM
  Halt Protease Inhibitor (ThermoFisher): 10 μL/mL
(2) In an Eppendorf Safe-Lock tube, layer 0.5 mL of 0.5 mm glass disruption beads (RPI). Add resuspended pellet to the top of the beads, and layer with another 0.5 mL of beads.
(3) Lyse in Bullet Blender Tissue Homogenizer, chilled with dry ice, for 5 min on speed 12.
(4) Add an additional 50 μL of lysis buffer to lysate and beads, and vortex to mix.
(5) Remove lysate from beads and transfer to a new microfuge tube, and centrifuge at 4 °C for 10 min at 15,000 RCF to clear lysate.
(6) Transfer supernatant to new microfuge tube.
(7) Quantify protein using a Qubit fluorometer protein assay (Invitrogen) or other suitable quantification method.

Western blotting:

(1) To 25 μg of protein, add an equal amount of 2× Laemmli buffer and boil samples at 95 °C for 5 min.
(2) Load samples onto a Bio-Rad Mini-PROTEAN TGX Stain-free gel, 4–15%.
(3) Run the gel for 5 min at 50 V.
(4) Increase the voltage to 120 V and run until the dye front reaches the bottom of the gel.
(5) Remove gel and image total protein using a Bio-Rad GelDoc.
(6) Transfer using Bio-Rad Trans-Blot Turbo as per manufacturer’s instructions.
(7) Block for 5 min in Bio-Rad EveryBlot blocking buffer.
(8) After 5 min of blocking, add α-puromycin antibody (catalog no. MABE343; Millipore) at a 1:1000 dilution.
(9) Incubate overnight at 4 °C.
(10) Wash the blot with Tris-buffered saline containing 0.5% Tween 20 (TBST) for 5 min (repeat ×3).
(11) Incubate with HRP-conjugated α-mouse secondary antibody (catalog no. 7074S; Cell Signaling Technologies) at a 1:10,000 dilution.
(12) Wash the blot with TBST for 5 min (repeat ×3).
(13) Apply chemiluminescent substrate to the blot and image using Bio-Rad ChemiDoc.
(14) Quantify total signal for each lane and normalize to total protein for each respective lane.

This method for assessing translational output does not come without drawbacks, chief among which is that upon incorporation of puromycin into the nascent polypeptide chain, the polypeptide is released prematurely into the cytosol, resulting in a detrimental accumulation of protein products. Additionally, this technique is particularly sensitive to the final concentration of puromycin used and the time allowed for incorporation, so great care needs to be taken to achieve accurate results. Typical concentrations of puromycin used in these assays range from 1–10 μg in mammalian cells and *S. cerevisiae*. However, application of this technique to *C. neoformans* required an increased puromycin concentration. It is unclear if this is due to decreased cell permeability to the drug, or if there is some alternative metabolism. We have previously shown that our optimized protocol demonstrated reduction in puromycin incorporation following treatment with hydrogen peroxide, supportive of strong translational repression that was also seen by polysome profiling [10].

#### 2.3.2. Measuring Translational Output using Click Chemistry

A more recent technique to emerge in measuring translational output is through incorporation of the methionine analog homopropargylglycine (HPG). When grown in methionine-free media, *C. neoformans* will incorporate HPG into the nascent polypeptide chain at methionine codons in actively translating cells. HPG contains an alkyne moiety, which can be labeled with an azide moiety containing dye through a click chemistry reaction [54]. After conjugation of the fluorophore, the fluorescent signal from the labeled polypeptide chains can be visualized by fluorescence microscopy or quantified using flow cytometry, resulting in a readout of translational output during the time in which the cells were treated (Figure 3B). Unlike with puromycin, the incorporation of HPG does not result in release of the nascent peptide, allowing translation to continue uninterrupted, providing a more accurate assessment of translational output, and its incorporation is dependent on ribosome translocation, rather than an empty A site.

Assessing translational output using click chemistry:

(1) Start 50 mL cultures in baffled flasks at OD_600_ = 0.15–0.20 in YNB-2% Dextrose (yeast nitrogen based without amino acids)*. Incubate cultures shaking until mid-logarithmic growth phase is reached, an OD_600_ = 0.55–0.70. *Media used for HPG incorporation must be methionine-free.
(2) If translation is to be assessed for response to stress or specific compounds, treat cultures appropriately ensuring a no-stress/no-drug control is also analyzed.
(3) 10 min prior to your desired time point, aliquot 1 mL of treated cells into a microfuge tube and add HPG to a final concentration of 50 mM.
(4) Allow HPG to incorporate for 20 min.
(5) Pellet cells by centrifuging for 2 min at 4000 RPM and quickly resuspend in 1 mL of ice cold 70% ethanol.
(6) Fix overnight at 4 °C.
(7) Carry out the click chemistry labeling reaction using Invitrogen’s Click-iT Protein Reaction Buffer Kit (catalog number: C10276) as per manufacturer’s instructions
(8) Quantify fluorescent signal by fluorescence microscopy, normalizing to the nuclear mask stain, or by flow cytometry.

## 3. Conclusions

The regulation of translation is critical for *C. neoformans* during host adaptation. Through our studies of the translational response to stress, we seek to understand the use of conserved mechanism and identify novel features of the *C. neoformans* translational response. Understanding these translational mechanisms, which allow for this adaptation, presents valuable avenues for discovering novel treatments against *C. neoformans.*
